# Does Male Circumcision Prevent HIV Infection?

**DOI:** 10.1371/journal.pmed.0020393

**Published:** 2005-10-25

**Authors:** Nandi Siegfried

## Abstract

Siegfried discusses the first reported randomized controlled trial of whether circumcision protects against HIV, published in *PLoS Medicine*.

Given the devastating mortality and morbidity associated with HIV/AIDS, many potential prevention measures against HIV infection have been explored. Male circumcision is one of these, and seven reviews of the literature [1–7], including two meta-analyses [4,5], have been published. However, as pointed out in the Cochrane systematic review of the subject, all studies to date were observational, and many were of poor quality. In the absence of any experimental evidence, no causal relationship between male circumcision and HIV prevention could be confidently assumed [7].

## The First Randomised Trial

In this issue of *PLoS Medicine*, Auvert and colleagues report results from the first completed trial of male circumcision for reducing HIV infection in South African heterosexual men [8]. The authors conducted a randomised and blindly evaluated trial in a semiurban area near Johannesburg in which the background HIV prevalence rate among heterosexual men was 4.4%. Between July 2002 and February 2004, they randomised 3,274 men, with 1,617 undergoing medical circumcision at the beginning of the trial (the intervention group) and 1,657 remaining uncircumcised (the control group). The men were followed up at three clinic visits at 3, 12, and 21 months, and were tested for HIV at each visit.

At an interim analysis done after all participants had completed the 12-month clinic visit, the Data Monitoring and Safety Board stopped the trial on the basis of the interim results. The results showed that, after excluding those men who were HIV-positive at the beginning of the trial (*n* = 146), 20 of the circumcised men became infected with HIV during the trial compared with 49 men in the control group. The risk of acquiring HIV infection was significantly reduced by 60% in the men who had undergone circumcision (incidence rate ratio = 0.4; *p* < 0.001). All men in the control group were then offered circumcision.

## Strengths of the Study

One strength of this study is that participants were drawn from the general population, increasing the generalisability of the findings. In addition, the relatively low loss to follow-up (7.9% overall) demonstrates that trials of this nature can be adequately conducted in poorly resourced settings.

The researchers, the participants, and their supporters should be congratulated for attempting and successfully completing a trial as complex as this. The interim analysis was planned, and used an appropriate statistical stopping rule, thus reducing the chance of randomly exaggerated treatment effects [9].

## Concerns about the Randomisation

The researchers did not report how the randomisation sequence was generated, and they used an unusual form of allocation to comparison groups. As participants requested to be actively involved in the allocation process, they were invited to choose an envelope from a pre-prepared box of ten envelopes. After each allocation, this box was then refilled with envelopes from another box containing five envelopes for the intervention group and five for the control group. Although the envelopes were equally distributed in the second box, this was not necessarily the case with the participant box. This unequal distribution partly explains why the numbers in the comparison groups differ by a total of 40.

Perhaps of more concern is that allocation concealment may have been inadequate given that the centre manager was responsible for filling both boxes of envelopes, and potentially could have subverted the treatment allocation, thus introducing selection bias. Inadequate allocation concealment has been shown to be associated with exaggerated treatment effects [10,11]. Despite this, as the baseline characteristics of both comparison groups are similar and the sensitivity analyses of the results are robust, the effect of these quality parameters is probably negligible.

## Ethical Concerns

Although it is unlikely to have affected the results, the trialists decided not to inform participants of their HIV status, neither at the beginning or end nor during the trial. The authors argue that at the time of the trial, antiretrovirals were unavailable in the public health sector in South Africa, and that the participants received intensive counselling on how to avoid contracting and transmitting HIV. The researchers chose not to inform those who were HIV-positive at the beginning because they were concerned that exclusion from the trial would be stigmatising. Men were encouraged to attend voluntary counselling and testing (VCT) in a nearby public clinic or in a special VCT centre in the same building as the investigation centre. Men who tested positive for other sexually transmitted infections that were treatable locally were referred to the local clinic.

Avoiding stigmatisation is an important consideration, but it could be argued that if withholding HIV status was the only feasible option, then the conditions were not suitable to conduct such a trial. In fact, referring participants to the local VCT centre or the clinic for treatment of other sexually transmitted infections may have resulted in more stigmatisation, as this referral would have been visible to others rather than only to those within the trial processes. Some might argue that the nondisclosure of HIV status fails the test of beneficence (the obligation to prevent and remove harms and to promote the good of a person by minimising the risks incurred to the research participant and maximising the benefits to them and others). Not only were participants affected by nondisclosure, but so were their partners. It is unlikely that this approach would be tolerated in a more developed setting [12].

## Policy Implications

The trialists suggest that circumcision could be rapidly incorporated into national plans of countries where circumcision is not widely practised, while recognising that promotion of circumcision may also lead to undesirable outcomes such as undermining condom promotion. They are right to argue that we need to seriously consider circumcision as a potential prevention method, but it seems wise to be more cautious in making recommendations for policy. Within- and between-country differences in culture, religion, and social norms will need to be very carefully considered before implementing circumcision programmes. Crucially, the results of two additional trials underway in Uganda and Kenya are awaited. Considering the results of all three trials together is likely to provide us with stronger evidence to guide policy.

## Abbreviation

VCT: voluntary counselling and testing

## References

[pmed-0020393-b1] Moses S, Plummer FA, Bradley JE, Ndiya-Achola JO, Nagelkerke NJD (1994). The association between lack of male circumcision and risk for HIV infection: A review of the epidemiological data. Sex Transm Dis.

[pmed-0020393-b2] DeVincenzi ID, Mertens T (1994). Male circumcision: A role in HIV prevention?. AIDS.

[pmed-0020393-b3] Moses S, Bailey RC, Donald AR (1998). Male circumcision: Assessment of health benefits and risks. Sex Transm Dis.

[pmed-0020393-b4] Howe RV (1999). Circumcision and HIV infection: Review of the literature and meta-analysis. Int J STD AIDS.

[pmed-0020393-b5] Weiss HA, Quigley MA, Hayes R (2000). Male circumcision and risk of HIV infection in sub-Saharan Africa: A systematic review and meta-analysis. AIDS.

[pmed-0020393-b6] Bailey RC, Plummer FA, Moses S (2001). Male circumcision and HIV prevention: Current knowledge and future research directions. Lancet Infect Dis.

[pmed-0020393-b7] Siegfried N, Muller M, Deeks J, Volmink J, Egger M (2005). HIV and male circumcision—A systematic review with assessment of the quality of studies. Lancet Infect Dis.

[pmed-0020393-b8] Auvert B, Taljaard D, Lagarde E, Sobngwi-Tambekou J, Sitta R (2005). Randomized, controlled intervention trial of male circumcision for reduction of HIV infection risk: The ANRS 1265 trial. PLoS Med.

[pmed-0020393-b9] Schulz KF, Grimes DA (2005). Multiplicity in randomised trials II: Subgroup and interim analyses. Lancet.

[pmed-0020393-b10] Schulz KF, Chalmers I, Hayes RJ, Altman DG (1995). Empirical evidence of bias. Dimensions of methodological quality associated with estimates of treatment effects in controlled trials. JAMA.

[pmed-0020393-b11] Kunz R, Vist G, Oxman A (2002). Randomisation to protect against selection bias in health-care trials. Cochrane Database Syst Rev.

[pmed-0020393-b12] Clark P (2002). AIDS research in developing countries: Do the ends justify the means?. Med Sci Monit.

